# Supplementary material for: Content-rich biological network constructed by mining PubMed abstracts
Source: BMC Bioinformatics. 2004 Oct 8;5:147. doi: 10.1186/1471-2105-5-147 (PMC528731; doi:10.1186/1471-2105-5-147)
Supplement: Additional File 5 — The original Chilibot query results of the term "long-term potentiation (LTP)" and 22 other terms, limiting the latest references analyzed to the years 1990, 1995, 2000, and 2004. [file 1471-2105-5-147-S5.bz2 › chilibotAdditionalFile5/ltp2000/html/PKA_NMDA.html]

 


 **PKA** and **NMDA** 
  
Found 68 abstracts in PubMed,  **30 abstracts were retrieved and analyzed**.  


---

 Search Google  |
 PDF files only 
|  EDU domain only 

---

**Interactive relationship** (e.g. stimulation, inhibition, etc)

- The  **NMDA**  receptor subunits are directly phosphorylated at specific sites by protein kinase A  **PKA** , resulting in the activation of  **NMDA**  receptor mediated excitatory postsynaptic currents.  Ref: 10623586 Biochem Biophys Res Commun, 2000
- ...  **PKA**  activation overcame constitutive PP1 activity and conferred rapid enhancement of  **NMDA**  receptor currents.  Ref: 10390370 Science, 1999
- In contrast, stimulation of  **NMDA**  receptors led to an enhancement of CKII and  **PKA**  dependent phosphorylation only in slices of young animals.  Ref: 10226777 Hippocampus, 1999
- There was no developmental change in the time course of phospho CREB induction that followed KCl depolarization or  **PKA**  activation, nor was there a developmental change in the time course of phospho Erk1 2 induced by  **NMDA**  receptor activation.  Ref: 10804193 J Neurosci, 2000
- Yotiao, an  **NMDA**  receptor associated protein, bound the type I protein phosphatase PP1 and the adenosine monophosphate cAMP dependent protein kinase  **PKA**  holoenzyme.  Ref: 10390370 Science, 1999
- It is suggested that activation of  **PKA**  by D2 receptor blockade leads to  **NMDA**  receptor phosphorylation in the dendrites or phosphorylation of transcription factors in the nucleus.  Ref: 11102574 Brain Res, 2000

**Parallel relationship** (e.g. studied together, co-existance, homology, etc.)

- The  **NMDA**  receptor mediated component of excitatory synaptic currents was also inhibited by PDGF, and this inhibition was prevented by co application of a  **PKA**  inhibitor.  Ref: 10521446 J Biol Chem, 1999
- **NMDA**  induced currents recorded from XENOPUS oocytes injected with cRNA encoding the  **NMDA**  receptor subunits NR1, NR2A, and or NR2B, mainly found in rat hypothalamus, were not affected by  **PKA**  activation but were increased by protein kinase C PKC stimulation.  Ref: 10899951 J Neurochem, 2000
- To increase levels of  **PKA**  activation to the extent that  **NMDA**  receptor blockade would have less effect on eticlopride mediated gene induction in all regions of striatum, we administered the phosphodiesterase inhibitor IBMX to animals treated with eticlopride.  Ref: 11102574 Brain Res, 2000
- Preinjection of oocytes with calyculin A impeded the  **PKA**  but not the PKC mediated potentiation of hypothalamic  **NMDA**  induced currents.  Ref: 10899951 J Neurochem, 2000
- Less  **PKA**  activation by the low dose of eticlopride might explain why the expression was more sensitive in the lateral striatum to  **NMDA**  receptor blockade than in our previous study.  Ref: 11102574 Brain Res, 2000
- For glutamate receptors, non  **NMDA**  receptor phosphorylation by  **PKA**  is stimulated by dopamine, while  **NMDA**  receptor phosphorylation by  **PKA**  and PKC can be induced via the activation of beta adrenergic receptors, and metabotropic glutamate or opioid receptors, respectively.  Ref: 10218114 Adv Second Messenger Phosphoprotein Res, 1999
- Ten minutes prior to the retention test, through indwelling cannulae placed in the CA1 region of the dorsal hippocampus, they received 0.5 microl infusions of saline, a vehicle 2% dimethylsulfoxide in saline, the glutamate  **NMDA**  receptor blocker, aminophosphonopentanoic acid AP5 5.0 microg, the AMPA kainate receptor blocker, cyanonitroquinoxaline dione CNQX 0.25 or 1.25 microg, the metabotropic receptor antagonist, methylcarboxyphenylglycine MCPG 0.5 or 2.5 microg, the inhibitor of calcium calmodulin dependent protein kinase II KN62 3.5 microg, the inhibitor of cAMP dependent protein kinase  **PKA** , Rp cAMPs 0.1 or 0.5 microg, the stimulant of the same enzyme, Sp cAMPs 0.1 or 0.5 microg, or the inhibitor of the mitogen activated protein kinase MAPK kinase, PD098059 10 or 50 microM.  Ref: 10840135 Behav Brain Res, 2000
- Hence, yotiao is a scaffold protein that physically attaches PP1 and  **PKA**  to  **NMDA**  receptors to regulate channel activity.  Ref: 10390370 Science, 1999
- This effect depends upon an inhibitory feedback action of adenosine, liberated following facilitation of postsynaptic  **NMDA**  receptors by D1 receptor activation, an action independent of adenylyl cyclase stimulation or cyclic AMP dependent protein kinase  **PKA** .  Ref: 10218863 Neuropharmacology, 1999
- Application of forskolin, which activates  **PKA**  by means of cyclic AMP stimulation, caused a transient increase of  **NMDA**  induced currents.  Ref: 10899951 J Neurochem, 2000
- In recordings from inside out patches, the catalytic fragment of  **PKA**  did not itself alter  **NMDA**  single channel activity, but it blocked the up regulation of these channels by a Src activator peptide.  Ref: 10521446 J Biol Chem, 1999
- Thus, PDGF receptors depress  **NMDA**  channels through a calcium and  **PKA**  dependent inhibition of their modulation by c Src.  Ref: 10521446 J Biol Chem, 1999
- Hence, A2A receptors appear to negatively modulate  **NMDA**  receptor channel conductance via the phospholipase C inositoltriphosphate calcium pathway rather than the adenylate cyclase  **PKA**  pathway.  Ref: 9871438 Amino Acids, 1998
- In the present study we investigated the modulation of hypothalamic  **NMDA**  receptor mediated currents by cyclic AMP dependent protein kinase  **PKA**  using the two electrode voltage clamp technique in XENOPUS oocytes injected with rat hypothalamic mRNA.  Ref: 10899951 J Neurochem, 2000
- They require metabotropic and or  **NMDA**  glutamate receptors and  **PKA**  and MAPK activity.  Ref: 10996059 Behav Brain Res, 2000
- Thus, the levels and cellular localization of activated  **PKA**  may determine if D2 antagonist mediated gene expression is dependent on  **NMDA**  receptor activation.  Ref: 11102574 Brain Res, 2000
- Further, chronic... treatment of cells with either a protein kinase inhibitor H 7 or a cAMP dependent protein kinase  **PKA**  inhibitor H 89 abolished this effect of sulpiride on the  **NMDA**  induced calcium i changes.  Ref: 10025680 Synapse, 1999
- The amnesic effect of novelty appears to rely on N methyl D aspartate  **NMDA**  receptor and calcium calmodulin dependent protein kinase II CaMKII dependent... aspects of its habituation learning.  Ref: 10510197 Eur J Neurosci, 1999
- Here, we provide evidence for the participation of hippocampal  **PKA**  CREB and MAPK Elk 1 pathways, via activation of  **NMDA**  receptors, in memory formation of a one trial avoidance learning in rats.  Ref: 10719213 Brain Res Mol Brain Res, 2000
- Irrespective of the cultivation time, survival promoting effects of both BDNF and NT 3 on dopaminergic neurons were induced or potentiated upon simultaneous depolarization of cultured mesencephalic cells with  **NMDA**  or upon activation of cAMP  **PKA**  dependent signaling pathways with dibutyryl cAMP.  Ref: 10964491 Exp Neurol, 2000
